# Advancing Nursing Education: Evaluating Artificial Intelligence (AI) and Escape Room (ER) Approaches for E‐Learning and Interpretation Skills

**DOI:** 10.1155/nrp/8869482

**Published:** 2026-04-10

**Authors:** Azin Salimi, Masoumeh Nouri, Amirreza Moradi, Alireza Vasiee

**Affiliations:** ^1^ Student Research Committee, Department of Nursing, Faculty of Nursing and Midwifery, Ilam University of Medical Sciences, Ilam, Iran, medilam.ac.ir; ^2^ Department of Nursing, Faculty of Nursing and Midwifery, Ilam University of Medical Sciences, Ilam, Iran, medilam.ac.ir

**Keywords:** artificial intelligence, educational technology, gamification, nursing, nursing education

## Abstract

**Introduction:**

The spread of new educational technologies has challenged the cold and inflexible framework of traditional education, aiming to replace centuries‐old methods with short, low‐cost methods to improve learning and educational productivity. Thus, this study aimed to compare the effects of artificial intelligence (AI) and escape rooms (ERs) on e‐learning satisfaction and the skill of arterial blood gas (ABG) interpretation in nursing students.

**Materials and Methods:**

The current study was conducted as a quasi‐experiment on 91 nursing students in the third to sixth academic semesters, with a convenient sampling approach to AI, ER, and control groups by balanced block randomization. In the AI group, participants used ChatGPT; the ER group used the Twine platform; and the control group used the traditional method once a week. The E‐learning satisfaction scale (ELSS) and arterial blood gas interpretation scale (ABGIS) were filled before and two weeks after the end of interventions. Data were analyzed using descriptive and inferential statistical tests in SPSS V.25 at a 0.05 significance level.

**Results:**

In comparison to ER and control, AI significantly enhanced e‐learning satisfaction and ABG interpretation (*p* < 0.001). In terms of content, interface, and communication satisfaction, AI demonstrated superiority over ER (*p* < 0.001). The intervention made a big difference—explaining nearly 90% of the changes in ELSS and about 40% in ABGIS. Among all the factors, AI stood out the most, driving major improvements in both learning outcomes and student engagement.

**Conclusion:**

The use of AI compared to the educational ER and conventional methods significantly increases the learning of ABG interpretation and satisfaction with e‐learning. It is suggested that the positive and effective capabilities of AI be used as an educational assistant to enhance learning and teaching experiences in medicine and nursing.

## 1. Introduction

Education has been significantly affected by artificial intelligence (AI), a technology of the modern age that is tremendously transformative [1]. With the use of sophisticated algorithms and systems that analyze data, draw conclusions, and facilitate informed decision‐making, AI has created new opportunities for improving instructional strategies [2]. AI has been gaining more attention in recent years as it is used in schools [3]. It can personalize learning, provide instant feedback, and make teaching more efficient overall [4]. One of the most significant applications of AI in education is via adaptive learning systems. Lessons are modified to meet the requirements and skills of each individual learner [5]. AI‐powered chatbots act as virtual tutors, helping students with their questions. Automated assessments, educational simulations, and the combination of virtual and augmented reality make learning more interesting and effective [6]. These new technologies could change the way teachers do things, making learning more interactive and focused on the students [7]. Modern online education is developing with creative features such as gamification, augmented reality, simulators, interactive podcasts, and educational escape rooms (ERs) [8]. Also, With the expansion of technology in the last decade, its use to enrich learning and educational skills has created more user‐friendly approaches called educational ERs [9], compared to more traditional and limited educational methods in which the individual repeats different aspects of an educational subject many times after initial learning and feedback is given to him even without the presence of the instructor [10]. In addition, students use that opportunity to deal with problems and difficulties related to their coursework [11], which increases their engagement and enjoyment of the learning process, especially in the heavy educational courseworks [12, 13].

Educational ERs boost cognitive engagement, critical thinking, and problem‐solving [14]. Arterial blood gas (ABG) interpretation is a fundamental skill in healthcare especially in the field of nursing and medicine [15]. The ABG analysis gives fundamental data of a patient’s oxygenation, ventilation, and acid–base status that are necessary for the diagnosis and management of respiratory as well as metabolic disorders [16]. But limitations abound with the traditional way ABG interpretation is still taught in many educational programs that rely heavily upon less synergy approaches, essentially one‐way lectures and book learning exercises [17]. Those methods generally fall short of engaging students, and they are too static and especially lack that element of theory being translated into real‐world application. Therefore, students may be left with only partial understanding of the ABG interpretation or the application in practical protocols. In general, they require new directions to the educational challenges, focusing on active learning and engagement and education grounded in the real world [18].

Contemporary approaches like gamification, simulations, and experiential learning are not only far more effective than traditional techniques but also provide the students with content confidence to ultimately learn this core skill in a much more effective and sustainable manner [19]. Educational ERs become even more effective learning tools when combined with AI [20]. AI can help students make decisions during problem‐solving tasks, offer immediate feedback, and adjust learning paths based on their performance [21]. Research indicates that this approach not only increases motivation but also improves critical thinking and problem‐solving abilities [20]. A critical factor in the success of any instructional method is student satisfaction [14, 22]. Understanding how digital technologies affect learning experiences and satisfaction has become more crucial as a result of their fast proliferation [22]. Studies indicate that integrating technology into education—particularly interactive tools such as AI‐based systems—can boost motivation and deepen conceptual understanding [23].

A number of elements, including user‐friendly design, pertinent instructional content, efficient learner–system interaction, and prompt feedback, influence how satisfied students are with technology‐assisted learning [24]. AI‐driven solutions, in particular, enhance satisfaction by offering personalized teaching and analyzing student performance [24]. Additionally, interactive settings like AI‐enhanced educational ERs have been found to improve learning experiences and increase student satisfaction [25]. Emerging interactive modalities, such as AI‐enhanced educational ERs, have demonstrated potential in enriching learning experiences and enhancing student satisfaction. As AI becomes more common in schools and the ability to read ABG levels is very important in clinical practice, it is important to look into new ways to teach that will help students learn these skills.

Thus, this study aimed to compare the effects of AI and ERs on e‐learning satisfaction and the skill of ABG interpretation in nursing students.

## 2. Materials and Methods

### 2.1. Study Design

This study was a quasi‐experimental study that was done from March 11 to May 17, 2025, at the School of Nursing and Midwifery of Ilam, Iran.

### 2.2. Setting and Sample

Using the convenience sampling method, the study participants were chosen from among the third to sixth academic semester nursing students at the School of Nursing and Midwifery of Ilam. The inclusion criteria were having a personal smartphone, being willing to participate in the research, being familiar with basic computer skills, attending the study full‐time, and receiving a score of less than 10 on the arterial blood gas interpretation scale (ABGIS). The exclusion criteria were being transferred to another university, having a history of working as a paramedic or performing student work in a hospital, failing a previous semester’s practical nursing unit specifically due to clinical skill deficiencies, taking part in related workshops or research within the previous three months, not completing the questionnaire, or withdrawing from the study. All subjects gave their written and informed permission after enrolling in the research.

### 2.3. Sample Size, Randomization, and Blinding

To determine the sample size a priori, power analysis was performed using G∗Power 3.1.9.7 software. Analysis was based on one‐way ANOVA (F‐test; fixed effects), with α = 0.05, power 0.80, and three study groups. Given the evidence from educational studies, a medium effect size (Cohen’s f = 0.35) was assumed [23]. The required sample size was estimated to be approximately 82 people. Taking into account a possible 10% attrition, the final sample size was 91 people. Also, they were randomly assigned to three groups: the AI group (*n* = 30), the ER group (*n* = 30), and the control group (*n* = 31).

In order to balance by academic semester, stratified randomization was considered, with the semester classification factor [3–6]. Within each stratum, balanced block randomization (block size = 6) was performed to evenly allocate individuals to the three groups. A statistician who was not part of the research team prepared the allocation list independently before the experiment began. The research team was unaware of the allocation sequence, which ensured that the groups had the same number of participants to avoid potential bias.

### 2.4. Measuring Tools, Validity, and Reliability

#### 2.4.1. Demographic Tool Questionnaire

Demographic variables entailed age, academic semester, gender, marital status, grade point average (GPA), daily mobile usage (DMU), and daily computer usage (DCU). The GPA was figured out by taking the average of all the grades in the courses that were finished. It was then put into three groups: 12–13.99, 14–16.99, and ≥ 17. Participants reported their DMU and DCU themselves, and these were divided into three groups based on how many hours they used them each day:  < 2 h, 2–4 h, and  ≥ 5 h.

#### 2.4.2. E‐Learning Satisfaction Scale (ELSS)

This tool was designed by Hwang et al. which has 17 items in three factors: content (8 questions), interface (5 questions), and communication (4 questions). The scores were based on the Likert scale from *not at all* (1) to *strongly agree* (5). It scores from 17 to 85 where higher scores mean a better e‐learning satisfaction level. In the first iteration of this tool, the content validity index (CVI) and content validity ratio (CVR) values were 0.94 and 0.90, respectively. The internal consistency (Cronbach’s alpha) of the initial version was 0.93 [26]. Persian CVI and CVR were assessed using Baltz and Wassel’s approach on 10 faculty members of the nursing and midwifery faculty, who translated, reviewed, and corrected the materials. Upon completing the requisite computations and adjustments, the CVI and CVR were determined to be 0.84 and 0.88, respectively. The reliability assessment used an internal consistency test (Cronbach’s alpha) conducted on 25 nursing internships, with a coefficient of 0.82.

#### 2.4.3. ABGIS

This instrument was prepared by the study team for testing ABG interpretation learning and it contained 20 questions with multiple choices that were awarded a point for each accurate response and a zero point for a bad answer or no answer. The questions on this tool were created using the chapters provided by the ABG interpretation guidelines, with a minimum score of 0 and a maximum score of 20. Before the test was given to participants, the CVR and CVI were evaluated using Baltz and Wassel’s methodology on ten faculty members from the Faculty of Nursing and Midwifery who had taught theoretical and practical nursing units for at least two semesters and who had certain characteristics that were later reviewed and updated. The use of ABGIS across 25 nursing internships produced a CVR of 0.81 and a CVI of 0.84 after the required computations and modifications were made. An internal consistency test (Cronbach’s alpha) was performed on 25 nursing internships as part of the reliability evaluation; the results showed a coefficient of 0.77.

### 2.5. Intervention

After capturing the ethics code, random allocation, and inclusion and exclusion criteria, the study commenced. Also, demographic tools, ELSS, and ABGIS were filled by samples in online sheet form. At the beginning of the intervention, each group was taught how to work with the research tool (AI and ER) for twenty minutes. In each group, four educational sessions were considered each lasting for forty‐five minutes. In the AI group, sessions were held on Saturdays, in the ER group, on Mondays, and in the control group, on Wednesdays.

In the AI group, ChatGPT‐4 was used, which was developed by OpenAI. Initially, the monitor screen was divided into two parts, with the right side of the monitor displaying the interaction space with the GPT robot and the left side of the screen displaying a 150‐word slide consisting of three sections: pathophysiology, clinical manifestations, and therapeutic measures [18, 27]. Each user had 10 min to ask the robot questions in Persian for each of the three sections (maximum 5 questions in each section) and take notes on what the robot said, and after each section was completed, move on to the next section and ask the next questions. At the end of the 30‐min period, another 15‐min period was set aside for each participant to answer 10 multiple‐choice questions and a clinical scenario designed to fit the content of that session. Those who answered correctly were given an incentive code, and the time was added to their next test time in the remaining sessions. This process continued until the end of the fourth session.

In the ER group, the Twine online open‐source platform was used. In this platform, four ABG‐related disorders were designed (respiratory acidosis and alkalosis and metabolic acidosis and alkalosis) [18, 27]. In each session, one mentioned disorder drawn by Twine in order to use one computer by each participant in the ER group in the school of nursing site room. At the top of the ER training environment, a forty‐minute period was designed for content training, which was displayed when each student entered with the same username from the beginning to the end of the study. In the first five minutes, an introduction to the relevant disorder was given, then a puzzle and four questions were posed within thirty seconds to enter the next stage. In the next stage, pathophysiology and symptoms were taught for fifteen minutes, and in order to move to the next stage, the individual had to answer the new puzzle and four relevant questions correctly. If the individual answered the questions and scenarios correctly in less time, an incentive code was displayed and recorded for him so that at the end of each session, his level of competition with other participants could be determined. In the next twenty minutes, therapeutic measures and nursing interventions were taught with clinical scenarios appropriate to each disorder. If he gave the correct answers to these scenarios, he would receive points, and if he did not answer the relevant answers and the training correctly within the specified time, he would fall behind the competition. Finally, after the 40‐min period, another competition was designed in the same environment using the Twine platform for five minutes, in which all members of the ER group answered 10 multiple‐choice questions in five minutes, and those who had the most correct answers in less time were introduced as the top winners of that session, and an incentive code was recorded and displayed for them.

**FIGURE 1:**
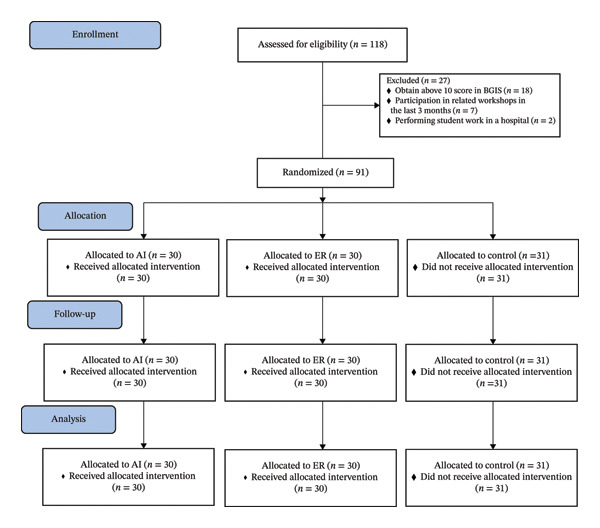
The process of allocating and implementing the study protocol according to control and intervention groups.

Participants in the control group were also given the same material that was provided to both groups in four online training sessions by the instructor who was not member of the research team using a PowerPoint presentation and lecture (traditional method) in Google Meet (for preventing any data leakage and awareness of students from each other). Lastly, the subjects filled out the ELSS and ABGIS again two weeks after the intervention ended without previous information (Figure 1).

### 2.6. Ethical Consideration

The acquisition of an ethics code (IR.MEDILAM.REC.1403.268), honesty in data reporting and library collection, signed informed permission from all samples in accordance with the proclamation of the Declaration of Helsinki, and interventional human principles were among the ethical concerns.

### 2.7. Statistical and Data Analysis

The variables were presented as mean, standard deviation, frequency, and percentage, and descriptive statistics were applied to the demographic data. Shapiro–Wilk (S‐W), chi‐square, post hoc Tukey HSD, and ANCOVA were among the analytical statistics tests. Effect sizes were calculated using Hedges’ g (bias‐corrected standardized mean difference) and interpreted using Cohen’s criteria (small (0.20–0.49), medium (0.50–0.79), and large (≥ 0.80)). The standard error was taken into consideration while analyzing the data using SPSS V.25.

## 3. Results

To check the normal distribution of the data, the S‐W test was implemented, and its results showed that all of variables have more than 0.05 which meant having normal distribution (Table 1).

**TABLE 1:** Normal distribution of quantitative variables among the groups.

Group	Variables									
	ABGIS		ELSS		ELSS subscales					
					Content		Interface		Communication	
	Before	After	Before	After	Before	After	Before	After	Before	After
AI	0.300	0.110	0.536	0.625	0.773	0.345	0.776	0.609	0.093	0.537
ER	0.364	0.737	0.088	0.215	0.373	0.116	0.524	0.145	0.471	0.829
Control	0.762	0.527	0.463	0.441	0.584	0.230	0.249	0.873	0.203	0.231

Abbreviations: ABGIS, arterial blood gas interpretation scale; AI, artificial intelligence; ELSS, E‐learning satisfaction scale; ER, escape room.

The demographic characteristics of participants were analyzed across the three groups: AI, ER, and control. Statistical analysis indicated no significant differences in age, academic semester, gender, marital status, GPA, DMU, or DCU among the groups (*p* > 0.05). This confirms that the baseline characteristics were comparable, and any observed differences in learning outcomes were likely due to the intervention itself (Table 2).

**TABLE 2:** Demographic characteristics of study participants among groups.

Demographic variables	Subcategories	AI (*N* = 30)	Escape room (*N* = 30)	Control (*N* = 31)	*p* value (chi‐square)
Age	18–20 years old	18 (60.0%)	11 (36.7%)	14 (45.2%)	0.229
	21–23 years old	5 (16.7%)	9 (30.0%)	10 (32.3%)	
	24 and higher	7 (23.3%)	10 (33.3%)	7 (22.6%)	

Academic semester	Third	6 (20.0%)	4 (13.3%)	11 (35.5%)	0.865
	Fourth	12 (40.0%)	7 (23.3%)	5 (16.1%)	
	Fifth	9 (30.0%)	6 (20.0%)	5 (16.1%)	
	Sixth	3 (10.0%)	13 (43.3%)	10 (32.3%)	

Gender	Male	14 (46.7%)	19 (63.3%)	23 (74.2%)	0.757
	Female	16 (53.3%)	11 (36.7%)	8 (25.8%)	

Marital status	Single	29 (96.7%)	24 (80.0%)	27 (87.1%)	0.901
	Married	1 (3.3%)	6 (20.0%)	4 (12.9%)	

Grade point average (GPA)	12–13.99	4 (13.3%)	5 (16.7%)	3 (9.7%)	0.242
	14–16.99	24 (80.0%)	20 (66.7%)	26 (83.9%)	
	17 and higher	2 (6.7%)	5 (16.7%)	1 (3.2%)	

Daily mobile usage (DMU)	Less than 2 h	3 (10.0%)	1 (3.3%)	1 (3.2%)	0.691
	2–4 h	8 (26.7%)	5 (16.7%)	12 (38.7%)	
	5 h and higher	19 (66.7%)	24 (46.7%)	18 (80.0%)	

Daily computer usage (DCU)	Less than 2 h	16 (60.0%)	13 (60.0%)	21 (53.3%)	0.532
	2–4 h	8 (26.7%)	12 (26.7%)	9 (26.7%)	
	5 hours and higher	6 (13.3%)	5 (13.3%)	1 (20.0%)	

AI had the highest improvements across all measures (ABGIS, ELSS, and its subscales). After intervention, the total score of ELSS significantly increased (57.20 ± 5.48, *p* < 0.001), and the ABGIS score nearly doubled (6.84 to 14.33) (*p* < 0.001) (Table 3). ER showed significant improvements, but its effect was smaller than AI. The total score of ELSS increased to 51.87 ± 5.45 (*p* < 0.001), and ABGIS improved to 11.60 ± 2.44 (*p* < 0.001). The control group showed minimal changes, with ELSS scores decreasing from 28.16 to 28.07 (*p* = 0.818) but ABGIS scores rising slightly from 6.86 to 9.67 (*p* < 0.001) (Table 3). ELSS’ subcomponents (content, interface, and communication) followed the same trend. AI showed the highest improvement in all ELSS’ subscales, particularly communication (16.47 ± 2.19) (*p* < 0.001) (Table 3). ER enhanced ELSS subscales, with the interface subscale (20.13 ± 2.89) showing the greatest improvement (*p* < 0.001). The control group achieved the lowest results, with minimal or nonsignificant changes in all subscales (interface: 9.24 to 10.13, *p* = 0.102; communication: 7.02 to 7.40, *p* = 0.925; and content: 12.41 to 10.53, *p* = 0.520) (Table 3). These findings indicate that AI substantially enhances both e‐learning satisfaction and ABG interpretation in comparison to ER and control approaches (Table 3).

**TABLE 3:** Descriptive statistics of BGIS and ELSS and its subscales among the groups before and after intervention.

Groups	Variables														
	ABGIS			ELSS			ELSS subscales								
							Content			Interface			Communication		
	Before	After	*p* value (paired *t*‐test)	Before	After	*p* value (paired *t*‐test)	Before	After	*p* value (paired *t*‐test)	Before	After	*p* value (paired *t*‐test)	Before	After	*p* value (paired *t*‐test)
	M ± SD	M ± SD		M ± SD	M ± SD		M ± SD	M ± SD		M ± SD	M ± SD		M ± SD	M± SD	
AI	6.84 ± 0.28	14.33 ± 1.84	**< 0.001**	28.05 ± 0.45	57.20 ± 5.48	**< 0.001**	12.40 ± 0.28	26.33 ± 5.70	**< 0.001**	9.33 ± 0.32	14.40 ± 2.79	**< 0.001**	6.91 ± 0.27	16.47 ± 2.19	**< 0.001**
ER	6.58 ± 0.30	11.60 ± 2.44	**< 0.001**	28.22 ± 0.49	51.87 ± 5.45	**< 0.001**	12.53 ± 0.26	20.20 ± 3.40	**< 0.001**	9.23 ± 0.33	20.13 ± 2.89	**< 0.001**	7.01 ± 0.31	11.53 ± 2.26	**< 0.001**
Control	6.86 ± 0.23	9.67 ± 2.85	**< 0.001**	28.16 ± 0.63	28.07 ± 2.81	0.818	12.41 ± 0.27	10.53 ± 1.45	0.520	9.24 ± 0.19	10.13 ± 2.55	0.102	7.02 ± 0.41	7.40 ± 2.22	0.925

*Note:* *p* value = paired *t*‐test; “before” and “after” indicate pre‐ and postintervention measurements. The significant values (*p* values) are bolded.

Abbreviations: ABGIS, arterial blood gas interpretation scale; AI, artificial intelligence; ELSS, E‐learning satisfaction scale; ER, escape room; M, Mean; SD, standard deviation.

AI and ER both made e‐learning a lot more fun than it was for the control group (*p* < 0.001; Hedges’ g = 5.45 and 0.72, respectively). AI was considerably more satisfied than ER (MD = −5.33, *p* = 0.010, Hedges’ g = 0.96), which implies that integrating AI enhanced the learning process better (Table 4). In ABG interpretation, AI significantly improved interpretation scores compared to both ER (MD = −2.73, *p* = 0.009, Hedges’ g = 1.25) and control groups (MD = −4.67, *p* < 0.001, Hedges’ g = 1.91) (Table 4). ER vs. control was not significant (MD = 1.93, *p* = 0.083, Hedges’ g = 0.72), indicating that the ER method did not substantially enhance interpretation skills (Table 4).

**TABLE 4:** Comparison of ELSS and ABGIS scores among groups with Hedges’ g.

Comparison	MD (final ELSS)	*p* value^∗^ (final ELSS)	95% CI (ELSS)	Hedges’ g (ELSS)	MD (final ABGIS)	*p* value^∗^ (final ABGIS)	95% CI (ABGIS)	Hedges’ g (ABGIS)
AI vs. control	−29.13	< 0.001	(−33.35, −24.92)	6.64	−4.67	< 0.001	(−6.81, −2.53)	1.91
AI vs. ER	−5.33	0.010	(−9.55, −1.12)	0.96	−2.73	0.009	(−4.87, −0.59)	1.25
ER vs. control	23.80	< 0.001	(19.59, 28.01)	5.45	1.93	0.083	(−0.21, 4.07)	0.72

*Note:* Hedges’ g: standardized effect size (effect sizes were interpreted according to Cohen’s benchmarks (small: 0.20–0.49; medium: 0.50–0.79; large:  ≥ 0.80)).

Abbreviations: ABGIS, arterial blood gas interpretation scale; AI, artificial intelligence; CI, confidence interval; ELSS, E‐learning satisfaction scale; ER, escape room; MD, mean difference.

^∗^Post hoc Tukey HSD.

In the content subscale, both AI and ER performed significantly better than the control group (*p* < 0.001; Hedges’ g = 3.78 and 3.67, respectively). Also, AI was significantly superior to the ER (*p* < 0.001, Hedges’ g = 1.29) (Table 5). In the interface subscale, both AI and ER showed significant improvements compared to the control group (*p* < 0.001; Hedges’ g = 1.58 and 3.63, respectively). However, AI demonstrated significantly better performance than the ER (*p* < 0.001, Hedges’ g = −1.99) (Table 5). In the communication subscale, both AI and ER had a positive impact on interactions and communication compared to the control group (*p* < 0.001; Hedges’ g = 4.06 and 1.82, respectively). But, AI also showed a significant improvement compared to the ER (*p* < 0.001, Hedges’ g = 2.19) (Table 5). On the whole, the AI significantly outperformed both the ER and the control group in content, interface, and communication satisfaction (*p* < 0.001). These findings suggest that AI integration enhances engagement, usability, and learning effectiveness beyond traditional mentioned methods (Table 5).

**TABLE 5:** Comparison of ELSS subscales among groups with Hedges’ g.

Comparison	MD (content)	*p* value^∗^	95% CI (content)	Hedges’ g (content)	MD (interface)	*p* value^∗^	95% CI (interface)	Hedges’ g (interface)	MD (communication)	*p* value^∗^	95% CI (communication)	Hedges’ g (communication)
AI vs. control	−15.80	< 0.001	(−19.28, −12.32)	3.78	−4.27	0.0003	(−6.71, −1.82)	1.58	−9.07	< 0.001	(−11.05, −7.09)	4.06
AI vs. ER	−6.13	< 0.001	(−9.62, −2.65)	1.29	5.73	< 0.001	(3.29, 8.18)	−1.99	−4.93	< 0.001	(−6.91, −2.95)	2.19
ER vs. control	9.67	< 0.001	(6.18, 13.15)	3.67	10.00	< 0.001	(7.55, 12.45)	3.63	4.13	< 0.001	(2.15, 6.11)	1.82

*Note:* Hedges’ g: standardized effect size (effect sizes were interpreted according to Cohen’s benchmarks (small: 0.20–0.49; medium: 0.50–0.79; large:  ≥ 0.80)).

Abbreviations: AI, artificial intelligence; CI, confidence interval; ER, escape room; MD, mean difference.

^∗^Post hoc Tukey HSD.

An ANCOVA analysis was done to figure out how the intervention affected the scores on ELSS and ABGIS. The three study groups were considered as independent variables, and the final scores were the results that were being assessed. To account for any baseline differences among students from different semesters—and to reduce the impact of potential confounding factors—the semester was also included in the model as a covariate. The intervention explained 89% of the variation in satisfaction (*η* ^2^ = 0.89), confirming its strong effect. AI significantly improved ELSS compared to the ER (*η* ^2^ = 0.23, *p* = 0.009) (Table 6). The AI had the highest impact (*η* ^2^ = 0.92, *p* < 0.001), demonstrating its superior effectiveness (Table 6). The intervention accounted for 41% of the variation in ABGIS (*η* ^2^ = 0.41), meaning the effect was strong but slightly less than ELSS. AI had a greater impact on ABGIS than the ER (*η* ^2^ = 0.26, *p* = 0.004) (Table 6). Again, AI showed the strongest effect compared to the control (*η* ^2^ = 0.92, *p* < 0.001) (Table 6). The findings indicate that both the AI and ER significantly enhanced both outcomes, but AI was more effective in ELSS and ABGIS than others.

**TABLE 6:** Pairwise comparisons of AI and ER among groups.

Outcomes	Comparison	F‐statistic	*p* value^∗^	Partial eta squared (*η* ^2^)
Final ELLS	AI vs. ER	7.90	**0.009**	0.23
	ER vs. control	225.51	**< 0.001**	0.89
	AI vs. control	332.24	**< 0.001**	0.92
	Overall ANCOVA	162.45	**< 0.001**	0.89

Final ABGIS	AI vs. ER	9.41	**0.004**	0.26
	ER vs. control	225.51	**< 0.001**	0.89
	AI vs. control	332.24	**< 0.001**	0.92
	Overall ANCOVA	14.13	**< 0.001**	0.41

*Note:* If *η* ^2^ is below 0.06, the effect of the intervention is weak, if *η* ^2^ is between 0.06 and 0.14, the intervention has a moderate effect, and if *η* ^2^ is above 0.14, the intervention has a strong effect. The significant values (*p* values) are bolded.

Abbreviations: ABGIS, arterial blood gas interpretation scale; AI, artificial intelligence; ELSS, E‐learning satisfaction scale; ER, escape room.

^∗^ANCOVA.

## 4. Discussion

The present study was designed to investigate the effect of AI and educational ERs on ABG interpretation skills and satisfaction with electronic learning. The findings of this study showed that the use of AI had better outcomes compared to educational ERs and traditional training.

AI, as a new educational technology, has been teaching learners personalized learning experiences in the last half decade in order to improve their educational needs. Within the domain of clinical science education—especially in nursing—AI has demonstrated significant progress in enhancing specific clinical competencies, such as the interpretation of ABG results [16] which is consistent with current research. Consistent studies such as those conducted by Glauberman et al. and Koukourikos et al. emphasize the role of AI‐driven platforms in enhancing clinical reasoning and decision‐making skills [28, 29]. These tools provide user‐based feedback and help them identify and address educational gaps by creating questions and considering the dimensions of the subject [29]. AI systems can simulate a variety of clinical scenarios, allowing students to practice and refine their skills in a safe and controlled environment [16]. This level of need recognition and user adaptation highlights AI as an assistant and even a coach that enhances critical thinking and learning [28]. AI is extremely effective since it can track each student’s progress and provide them individualized help along the way which makes sure that everyone receives the help they need to do well [30] as a consistent approach. This method not only helps students accomplish better but it also boosts their confidence and keeps them motivated to keep practicing [31]. Despite the many benefits seen in AI, this tool has limitations, two of the most important of which include dependence on data induced by the data center or bugs that occur in some analyses in this assistant, which creates educational and ethical challenges for researchers and learners which is inconsistent with the following research [30]. Additionally, while AI excels in enhancing individual skills, it may lack the capacity to foster the interpersonal and teamwork skills that are equally critical in nursing practice [31].

Educational ERs are a collaborative and challenging approach to teaching learners that focuses on learning, critical thinking skills, and solution selection. In these immersive events, people have to work together to do tasks and solve challenges in a certain period of time which is comparable to what nurses do for a living. Two consistent research studies done by Gutiérrez‐Puertas et al. and Valdes et al. have shown the effectiveness of ERs in improving teamwork, leadership, and communication skills among nursing students [32, 33]. ERs make learning fun by getting people to work together and participate which helps students acquire the soft skills they need to care for patients well [34]. ERs also provide students a chance to use what they have learned in class in a real‐world setting, which helps them connect what they have learned in class with what they will do in the real world. For instance, in an ER scenario, students could have to figure out what is wrong with a patient and treat them for a complicated medical issue. To do this, they would have to use what they know about pathophysiology, pharmacology, and nursing treatments. In addition to improving clinical science students’ educational experience, this approach boosts their self‐assurance in their capacity to handle difficult clinical situations [35]. However, there are several issues that ERs might have. Collaboration and critical thinking are their strong suits, but they could fall short when it comes to teaching specific clinical skills that need one‐on‐one instruction, like reading ABGs [10]. According to Lim et al., ERs can be fun and engaging, but they often fall short when it comes to tackling complex clinical tasks. Compared to AI, educational ERs have limited depth of learning due to the lack of feedback and do not manage learning and identify its level based on user needs [36]. Furthermore, the design and implementation of ERs require a lot of time and resources, which makes the design of clinical and educational scenarios challenging [36].

In general, blended learning approaches are among the comprehensive solutions that enrich education. According to the findings of a study, blended learning approaches create participation and interaction with scenarios that make blended learning necessary, such as AI and ERs [37]. According to a study, AI can be used to prepare hospital scenarios. The same scenarios can then be transferred to an educational ER to simulate a real‐world environment and create challenges. An AI‐based platform was used to prepare students for the ER activity by teaching them the necessary skills and clinical knowledge [38]. Also, the same scenarios can then be transferred to an educational ER to simulate a real‐world environment and create challenges. An AI‐based platform was used to prepare students for the ER activity by teaching them the necessary skills and clinical knowledge [39].

### 4.1. Limitation and Strengths

The strengths of the present study include extensive inclusion and exclusion criteria, random assignment, comparison of more than three learning methods, and sufficient sample size. The limited weaknesses of this study include the length of sessions, the short time to complete the instruments, and no full psychometric validation for ELSS and ABGIS.

## 5. Conclusion

The use of AI compared to the educational ER and conventional methods significantly increases the learning of ABG interpretation and satisfaction with e‐learning. It is suggested that the positive and effective capabilities of AI be used as an educational assistant to enhance learning and teaching experiences in medicine and nursing.

## Author Contributions

Conceptualization, methodology, formal analysis, writing–original draft preparation, writing–review and editing, supervision, and project administration: Alireza Vasiee. Validation: Azin Salimi and Masoumeh Nouri. Investigation: Alireza Vasiee and Amirreza Moradi.^,^ Resources, data curation, software, and visualization: Azin Salimi, Masoumeh Nouri, and Amirreza Moradi.

## Funding

This inquiry was the result of a project approved by the Ilam University of Medical Sciences under project number 3031. No financial support was received for this research.

## Conflicts of Interest

The authors declare no conflicts of interest.

## Data Availability

The data that support the findings of this study are available on request from the corresponding author. The data are not publicly available due to privacy or ethical restrictions.
